# First Report of the Peach Root-Knot Nematode, *Meloidogyne floridensis* Infecting Almond on Root-Knot Nematode Resistant ‘Hansen 536’ and ‘Bright's Hybrid 5’ Rootstocks in California, USA

**DOI:** 10.21307/jofnem-2019-002

**Published:** 2019-03-29

**Authors:** Andreas Westphal, Zin T. Z. Maung, David A. Doll, Mohammad A. Yaghmour, John J. Chitambar, Sergei A. Subbotin

**Affiliations:** 1 Department of Nematology, University of California, Riverside, CA, USA; 2 University of California Cooperative Extension, Merced and Bakersfield, CA, USA; 3 Plant Pest Diagnostic Center, California Department of Food and Agriculture, CA, USA

**Keywords:** California, *Meloidogyne floridensis*, Peach root-knot nematode, Resistance

## Abstract

In April-August 2018, samples of galled roots with rhizosphere soil were collected from almond orchards in Atwater, Merced County and Bakersfield, Kern County, California. Almond trees (*Prunus dulcis*) grafted on ‘Hansen 536’ and ‘Brights Hybrid^®^5’ (peach-almond hybrid) rootstocks showed strong symptoms of growth decline. Extracted root-knot nematodes were identified by both morphological and molecular methods as *M. floridensis*. *Meloidogyne floridensis* was initially found in Florida, USA, and has not been reported from any other states and countries. This is a first report of *M. floridensis* in California and outside of Florida.

The peach root-knot nematode, *Meloidogyne floridensis*, is recognized as an emerging pathogen of commercial peach production because of its capability to overcome root-knot nematode resistance in rootstocks. This nematode was first described in Florida where it was found in 16 counties (Brito et al., 2015; Brito pers.comm). Although it was reported to infect peaches in 1966, the peach root-knot nematode was only described as a new species in 2004 (Handoo et al., 2004). In Florida, *M. floridensis* infects peach in nurseries and orchards, and was also identified on other economically important crops and on weeds. The nematode is able to parasitize *Prunus* rootstocks ‘Nemaguard’, ‘Flordaguard’, ‘Guardian’, ‘Okinawa’, and ‘Nemared’, all resistant to *M. incognita*, *M. javanica*, and *M. arenaria*. (Sherman and Lyrene, 1983; Smith et al., 2015).

In April-August 2018, samples of galled roots with rhizosphere soil were collected from almond orchards in Atwater, Merced County and Bakersfield, Kern County, California. Almond trees (*Prunus dulcis*) grafted onto peach-almond hybrid rootstocks ‘Hansen 536’ and ‘Bright’s Hybrid^®^5’, both resistant to southern root-knot nematode (*M. incognita*) and Javanese root-knot nematode (*M. javanica*) and having ‘Okinawa’ and ‘Nemaguard’, respectively, in their parentage (McKenry et al., 2007) showed strong symptoms of growth decline (Fig. 1). Extracted root-knot nematodes were identified by both morphological and molecular methods as *M. floridensis* at the Plant Pest Diagnostic Center, California Department of Food and Agriculture. This detection marked the first report of this species in California and outside of Florida. Due to its resistance-breaking ability and potential for further dispersal from infected areas, the peach root-knot nematode could cause significant negative economic impact on almond production in California.

**Figure. 1:**
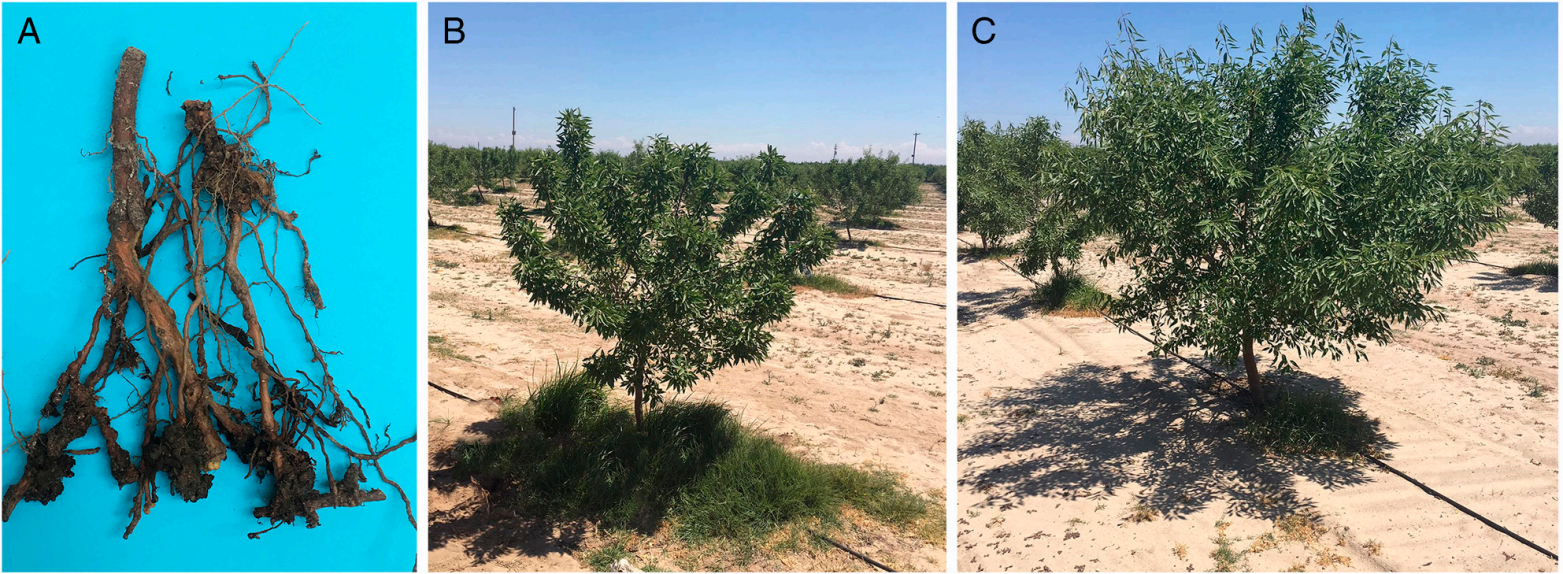
(A) Root galls on ‘Hansen 536’ rootstock of almond trees (*Prunus dulcis*) as scion; (B) Almond tree infected tree with *Meloidogyne floridensis*; (C) Almond tree, healthy.

For light microscopy, specimens were killed by gentle heat and fixed in 4% formaldehyde solution. Light micrographs were taken with an automatic Infinity 2 camera attached to a compound Olympus BX51 microscope equipped with Nomarski differential interference contrast. Morphometric mean, standard deviation and range values of second stage juveniles (J2s) included (n = 25): L = 374 ± 12.5 (357–405) μm; a = 26.1 ± 1.2 (22.8–28.0); b = 4.5 ± 0.3 (3.9–5.6); b’ = 3.0 ± 0.3 (2.6–3.7); c = 8.7 ± 0.7 (7.3–10.9); c’ = 4.5 ± 0.4 (3.6–5.4); labial width/labial height = 2.1 ± 0.4 (1.2–3.0) μm; stylet length = 14.1 ± 0.6 (13–15) μm; DGO = 3.3 ± 0.7 (2.0–4.8) μm; center of median bulb to anterior end = 55.1 ± 3.4 (50.8–67.6) μm; hyaline part of tail length = 8.4 ± 1.2 (5.2–10.4) μm and tail length = 42.8 ± 3.3 (34–51) μm. J2s had a smooth, truncated head that was slightly offset from the body by slight constriction, rectangular labial disc, four lateral longitudinal lines, and a tail tapering to a bluntly rounded terminus (Fig. 2). Males (n = 5): L = 1,219 ± 411 (675–1,725) μm; a = 40.7 ± 10.0 (25.7–52.8); b = 7.4 ± 2.4 (5.4–10.6); c = 159.3 ± 18.7 (146–172); stylet length = 20.0 ± 3.1 (17.5–23.8) μm; center of median bulb to anterior end = 88.8 ± 12.3 (80–97.5) μm; excretory pore to anterior end = 122.5 ± 9.0 (112.5–130) μm; spicules = 31.8 ± 4.8 (27.5–38.8) μm; gubernaculum = 6.3 μm. Body cylindrical, vermiform, tapering anteriorly; bluntly rounded to clavate posteriorly (Fig. 2). Perineal pattern with rounded dorsal arch, coarse broken to network-like striae in and above anal area, and faint lateral lines interrupting transverse striae (Fig. 2).

**Figure. 2:**
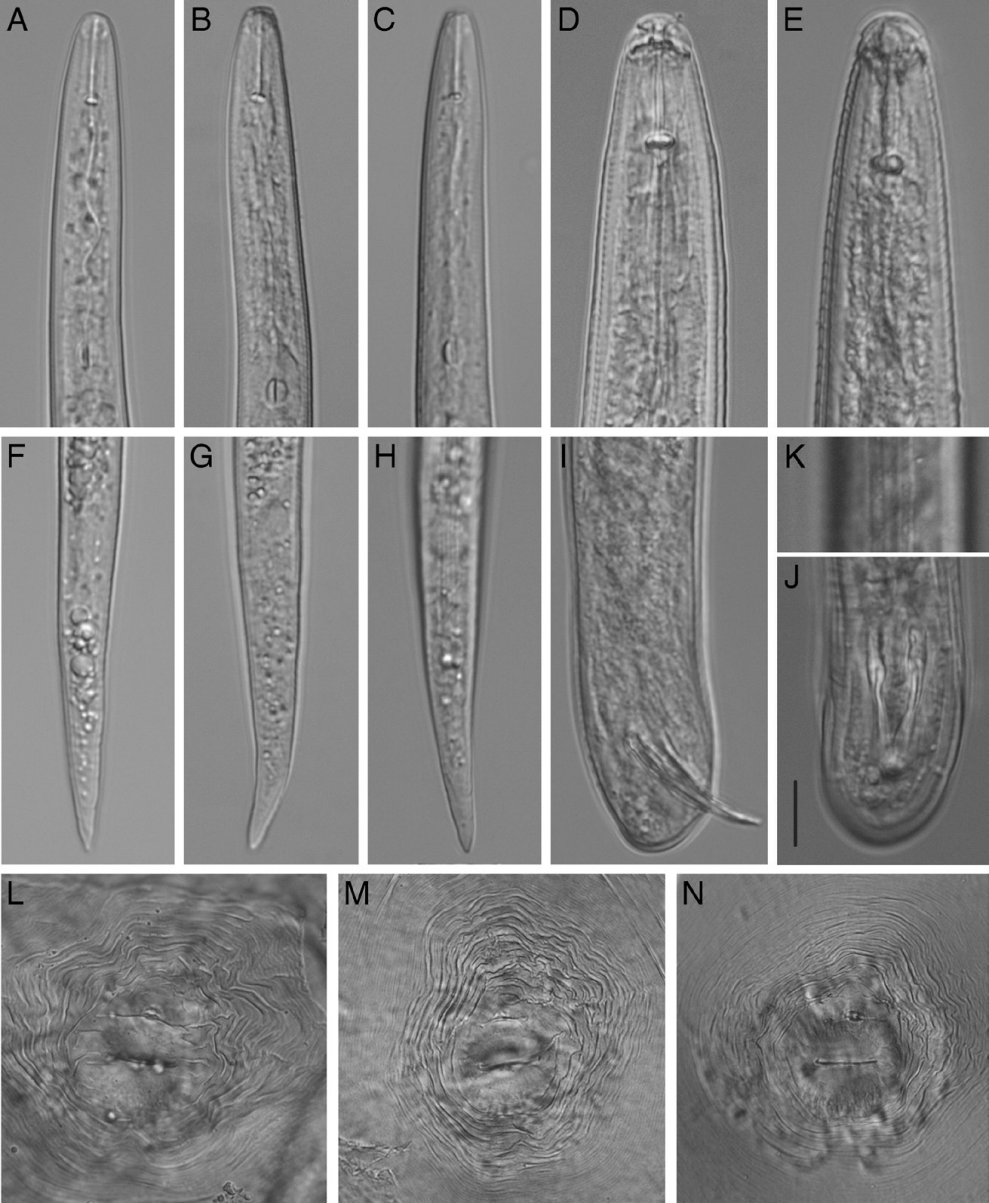
*Meloidogyne floridensis.* (A–C) Anterior region of J2s; (D and E) Head region of males; (F–H) Posterior region of J2s; (I and J) Posterior region of males; (K) Lateral field of male; (L–N) Perineal patterns of females. Scale = 10 μm for A–J and 20 μm for L–N.

Perineal patterns of females, body length, tail and hyaline tail lengths, and ratio values of J2s were consistent with those previously reported for isolates of this species from Florida, however, stylet length of the California population was notably longer (Handoo et al., 2004; Stanley et al., 2009).

DNA was extracted from 20 samples collected in Merced and Kern counties and containing 10 to 15 J2s each using the proteinase K protocol (Janssen et al., 2016). Two primer sets were used: forward NAD5F2 (5′-TAT TTT TTG TTT GAG ATA TAT TAG-3′) and reverse NAD5R1 (5′-CGT GAA TCT TGA TTT TCC ATT TTT-3′) for the amplification of partial *nad*5 gene (Janssen et al., 2016) and forward C2F3 (5′-GGT CAA TGT TCA GAA ATT TGT GG-3′) and reverse 1108 (5′-TAC CTT TGA CCA CTC ACG CT-3′) for amplification of the mtDNA region between *COII* and 16S rRNA (Powers and Harris, 1993). PCR products of these gene fragments were obtained and sequenced: MH729181, MH729182. Sequences of *nad*5 gene and fragment between *COII* and 16S rRNA genes were identical to the reference sequences of these genes published for *M. floridensis* by Janssen et al. (2016) and Smith et al. (2015), respectively. Morphological and molecular examination confirmed the species as *M. floridensis*. *Meloidogyne floridensis* was initially found in Florida, USA, and has not been reported in any other state or country. This is a first report of *M. floridensis* in California and outside of Florida.
